# Collectanea

**Published:** 1831-04

**Authors:** 


### COLLECTANEA.

Floriferis ut apes in saltibus omnia libant,
Omnia nos, itidem, depascimur aurea dicta.

### PATHOLOGY.

*Wound of the Head, and Loss of a large Portion of the Brain, followed by rapid Recovery.* Dr. Stefano was called, on the 31st of July, to a young man, named Pisano, æt. twenty-two, who had received, on the preceding evening, a severe blow from an axe upon the left parietal bone. The wound was extensive, and had been inflicted from behind, forwards, almost parallel with the sagittal suture, and about two fingers' breadth from it. The bones, and membranes of the brain, were cut through, and about three drachms of the cerebral substance had been lost; but still the intellectual functions were undisturbed, and remained unaffected throughout the progress of the case. The patient was occasionally troubled with a little bilious vomiting; the pulse was nearly natural. The edges of the wound were brought together; taking care, however, to leave an exit for fragments of bone, or pus, that must necessarily be secreted. The parts were covered with an emollient cataplasm, and a low diet and aperients were ordered. Towards evening, the pulse became hard and full, and a free bleeding was directed.

On the next day, the patient was again bled, and the aperient medicines were repeated. Portions of the brain, mingled with pus, continued to pass out through the part of the wound that had been left open.

On the third day, he was much better, and, as he had some appetite, a little milk was allowed. A free suppuration was now established, and several fragments of bone came away.

The wound improved in appearance daily; but, on the 13th of August, a medullary fungus had formed upon the injured part of the brain: it was prevented from increasing, by astringent applications. The patient was enabled, in three months from the time of the accident, to resume his laborious employment as an agricultural labourer.

This case forms an additional example of the powerful resources of nature. The slightest wounds of the head, an effusion of a few drops of blood within the cranium, often produce the most serious symptoms; whilst in this instance a very extensive wound, accompanied by a considerable loss of the cerebral substance, was not followed by any disturbance of the intellect, or affection of the head, and the cure proceeded almost with as much rapidity as if the wound had been slight and superficial.—*Osservatore Medico*, Novemb. 1830, No. 22.

---

### *Observations on Gaseous Tumors of the Uterus.*

*Acute Gaseous Tumor of the Uterus.* A woman, of full habit, in the prime of life, suddenly exposing herself to cold during menstruation, had the discharge cease. She immediately experienced a severe pain in the region of the uterus, and the size of that organ was considerably increased: it extended as high as the umbilicus, and on both sides to the groins. The patient was unable to move her legs, or even her body. The shivering, which happened at the commencement of the attack, was succeeded by fever and exacerbations; in the evening, thirst and restlessness. On examining the uterus with the finger, whilst the other hand was pressed on the fundus, a tympanitic sound was clearly distinguishable on percussion. A large quantity of blood was taken from the foot; emollient fomentations, and clysters of chamomile and elder flowers, were applied, without at all diminishing the size of the uterus: it even, indeed, assumed a rounder form. Leeches were applied to the vulva, with as little advantage. The intensity of the symptoms continuing to increase, another examination was made, by introducing the finger into the os uteri, and thus a vent was given to the discharge of fœtid gas.

From this time the size of the belly evidently diminished; but shortly after it again increased. Fumigations were introduced by means of a tube into the interior of the uterus, by which exit was given to a new flow of gas, still more abundant than the former, and accompanied by clots of blood. The patient remained in this state for some days, and completely recovered.

*Gaseous Tumor of the Uterus, resembling Pregnancy.* A woman, forty-two years of age, who had never borne children, had some symptoms of pregnancy. The menses, which had hitherto been regular, were suppressed, and the os uteri was completely closed; but she experienced none of those peculiar sensations which usually attend pregnancy: the uterus, however, towards the fifth month, was as high as the umbilicus, and, on slight pressure being made, it was possible to surround it completely with the hand. Such was the state of the patient, when, towards the end of the sixth month, all her hopes of pregnancy vanished; for, upon stooping down, a great quantity of flatus escaped from the uterus: the belly sank, and in a few days resumed its natural state.—*Opusculi della Societa Medico-Chirurgica di Bologna,* vol. iv.

---

### PRACTICAL MEDICINE.

*Use of Savin in Chronic and Passive Menorrhagia.* Dr. Wedekind strongly recommends this remedy in such cases; and, in the *Gemeinsame deutsche Zeitschrift für Geburtskunde,* vol. iv. cah. 4, Dr. Feist relates a case, in which the following formula was successfully used. The woman had had excessive menorrhagia for two years; she had no organic disease. ℞. Pulv. fol. Sabinæ ʒiij., Extract. Sabinæ ʒij., Ol. Sabinæ ℈i., to be divided into pills of three grains each. At first, three pills were taken each day, and the dose was gradually increased to ten daily. In seventeen days the hemorrhage ceased: the patient afterwards menstruated regularly.

We give this fact as we find it. In this country, savin has been chiefly used, not to arrest, but to excite hemorrhage from the uterus. Dr. Home, for example, says, that in five cases of *amenorrhœa,* which occurred at the Royal Infirmary of Edinburgh, four were cured by the sabina. He gave it in powder, from a scruple to a drachm twice a day. He thinks it well adapted for feeble patients, but deprecates its use in plethoric habits. Dr. Cullen says, that "its heating qualities always require a great deal of caution."

---

*Belladonna as a Preventive of Scarlet Fever.* The German physicians have for some time adopted the plan of giving small doses of belladonna, for the purpose of preventing the occurrence of scarlet fever in those persons who were exposed to the contagion. In our number for January 1828, p. 67, we mentioned some interesting facts connected with this subject. Dr. SAZYMA (Medicin Jahrbucher des k. k. cesterr. Staates, t. i. cah. 1, 1829, p. 116,) states that, out of sixteen children, to whom belladonna was given as a preservative from scarlatina, and all of whom were immediately exposed to the contagion, ten were completely protected. In four no eruption occurred, and the disease was confined to a slight sore throat. In two children scarlatina took place, although belladonna had been taken. In these six last cases, the medicine had been given too short a time to produce the desired effect.

---

### TOXICOLOGY.

*Cases of Poisoning by Cantharides.* By Mr. WILLIAMS, Surgeon, Bewdley.

CASE I. June 28th, about eleven o'clock P.M., I was called upon to attend William Glover, aged nineteen years, who complained of violent pain in his stomach, bowels, kidneys, and bladder; a burning heat in his throat, with a constant and most urgent desire to void his urine. He stated that, between three and four o'clock, he had taken, at the request of a young female, about half a pint of raspberry brandy, which was very thick, and soon after set out on an errand to Stourport: when on the road, felt violent pain in the stomach and bowels, which occasioned him frequently to lie down in the road, and caused him to be more than six hours in accomplishing the distance, which is only six miles. I went to the young female, who served in a liquor shop, and requested to see the bottle which contained the raspberry brandy: the bottle was promptly produced, and evidently had been recently washed; but the cork which stopped the bottle happened to be a very porous one, and in it were lodged many minute particles of the pulvis lyttæ. I immediately taxed her with having administered a deleterious drug to the young man, which she most resolutely denied, and attempted to account for the thickness of the brandy, and the young man's illness, from its having stood for two or three days in a copper vessel. I pointed out to her the flies collected in the cork, and ascertained afterwards that she had procured half an ounce of the Spanish flies from a druggist's shop that day; and there is every reason to believe that the greater part were administered to this and another young man. Having fully satisfied myself as to the cause of these urgent symptoms, I ordered him to drink freely of a mixture of sugar, water, and sweet oil, until vomiting be produced, which happened in about ten minutes; but I could not discern any particle of the flies in the ejected fluid. Ordered to drink freely of linseed tea.

June 29th, horâ four A.M. Skin became very hot; sense of burning in the throat more urgent; incessant straining to make water, which amounted to nothing more than a few drops of blood; pulse 100 per minute; the tongue put on the appearance of having been covered with a thick coat of drab-coloured paint. V.S. ad ℥xvi.; et capt. Ol. Ricini ℥iss. administ. enemata.

Horâ nine. Symptoms much the same; bowels not moved. Ordered the castor oil and clyster repeated.

Horâ twelve. Bowels acted upon twice: strangury still very distressing, nothing but a few drops of blood passing; great tenderness over the region of the kidneys, ureters, and bladder; pulse 100. V.S. ad ℥xiv.; repet. Ol. Ricini et enemata; take linseed tea and barley water freely.

Horâ five P.M. The tenderness had much increased, particularly over the region of the kidneys; great restlessness, with occasional delirium; pulse 115; other symptoms much the same. V.S. ad ℥xvi.: repet. Ol. Ricini et enemata.

Horâ twelve. Restlessness very great; constant delirium, with some slight convulsion; face much flushed; the conjunctiva charged with blood; strangury most urgent; pulse 125, hard, and not compressible; bowels were open. V.S. ad ℥xxiv.

*Note.* When I had taken away eighteen ounces, he became very faint; but I found there was such a disposition for reaction to take place, that I determined on taking away small quantities, so as to keep him in a state of syncope for nearly an hour: I then allowed him to lie down, and immediately gave him eight drops of laudanum in some linseed tea.

June 30th. Has had some sleep, and expresses himself much relieved; urine more copious, but very bloody, and attended with great pain in passing through the urethra; pulse ninety-six. Rept. Ol. Ricini et enemat. sextis horis. Bowels were acted on freely during the day, and in the evening felt better: has taken nothing but linseed tea and barley water.

July 1st. Has had some sleep; the urine voided amounted to eight ounces in twelve hours, very bloody; pulse ninety-six. Cont. Ol. Ricini et enemata.

July 2d. Bowels open; urine more copious and less bloody; pulse eighty-six. Cont. Ol. Ricini.

3d. In the same state as yesterday.

4th. Urine more copious and less bloody; pulse seventy. Capt. Ol. Ricini ℥i. omni mane.

5th, 6th, 7th. The oil was taken each morning, which acted daily on the bowels; the water was still a little tinged with blood, but was voided without pain. At this time the tongue, which put on a peculiar appearance at the commencement, now began to shew the papillæ, and in three days was quite clear, and the young man convalescent.

Case II. June 28th, on my return home from visiting William Glover, I was requested to see William Baylis, aged nineteen years, who stated that he had, at the request of the damsel in the dram shop, taken half a pint of raspberry brandy, about four o'clock that evening: in about half an hour afterwards, he became very faint, and vomited freely: he afterwards felt pain in his stomach and lower part of his bowels, with a frequent desire to void his urine. These symptoms had gradually become worse up to the time I saw him, when they were excessively urgent. On examining the ejected fluid, an immense quantity of the pulv. lyttæ was discoverable. Ordered him to drink largely of sugar, water, and olive oil, which soon caused him to vomit freely. He afterwards drank plentifully of linseed tea.

29th, horâ five A.M. Skin become hot; pulse ninety-six; great tenderness over the stomach, ureters, kidneys, and bladder; strangury very urgent; urine very bloody; tongue much coated. V.S. ad ℥xvi. Capt. Ol. Ricini ℥iss. administ. enemata.

Horâ ten. Bowels moved twice; considers himself easier; urine still scanty and bloody, and attended with great pain in passing through the urethra; pulse ninety.

Horâ six P.M. Tenderness much increased; strangury more urgent; pulse 110. V.S. ad ℥xv. Rept. Ol. Ricini et enemata.

Horâ twelve. Bowels acted on freely; pulse ninety-six; expresses himself much relieved.

June 29th. Has had some sleep during the night. The castor oil and enema were administered early this morning, and produced two copious evacuations: tenderness much less; strangury less urgent; urine more copious, but bloody; pulse ninety.

30th. Bowels open from taking castor oil; urine more copious, and less bloody. Ordered to take the castor oil night and morning; which he did for four days; at the end of which time the tongue became clean, and he convalescent.—*Midland Med. Rep.*

---

### SURGERY.

***Belladonna used with advantage in Constriction of the Anus.*** A man, forty years of age, had been troubled with piles for ten years, which sometimes gave rise to great pain, which was relieved by local and general bleeding, warm baths, and clysters. At length, however, the severity of his sufferings increased: the pain now extended from the anus to the abdomen, and was particularly acute in the right hypochondrium; bleeding had in a great measure lost its effect. The torment the patient suffered when he had a motion was excessive, especially if his bowels were rather constipated. Upon examination, a sinus was discovered near the anus, the division of which relieved the man, and he remained in good health for two years. At the expiration of this time, after a long journey on horseback, during the heat of summer, the pain in the anus returned; and now there was so much constriction of the part that the fæces were with much difficulty evacuated; a finger could hardly be introduced into the anus. Another sinus had formed, but, instead of dividing it as before, M. Laborderie introduced pledgets of lint, covered with extract of belladonna and acetate of lead. The pain was almost immediately relieved, and ceased entirely in a few days.

M. Laborderie concludes, therefore, that we must not too quickly determine upon the division of the sphincter in cases of fissures of the anus. Many experienced practitioners advise that the treatment of such cases should commence by employing means to dilate the part. The effect of the belladonna is not quite certain: in this case it was combined with the acetate of lead, which very frequently relieves pain in such instances, although too little dependence is usually placed in this remedy.—*Journal Complément.*

---

*Remarks on Amaurosis; illustrated with Cases.* By JAMES J. KNOX, A.M., Member of the Faculty of Physicians and Surgeons, Glasgow, &c.

Different opinions have been entertained regarding the nature of those changes produced on the retina by concussion, so as to occasion amaurosis. By some it has been supposed that extravasation of blood is the cause of the amaurosis; it is obvious, however, that this opinion is incorrect, as the effects of extravasation would be but temporary, whilst in general those of concussion are permanent and incurable. It is nearer the truth, I apprehend, to suppose paralysis of the retina the cause of the affection, as in general no morbid appearance is visible in the interior of the eye, and it is also exceedingly probable, as stated by the celebrated Beer, that rupture or laceration of that membrane may be produced from concussion of the eyeball.

*Prognosis.* The prognosis of amaurosis consequent to concussion of the eyeball, is always very bad. In most cases it will be found that vision is irrevocably destroyed, and that although we may apparently gain some advantage in restoring some of the parts, as the iris, to their natural colour, that vision is very little, if at all, improved. Nevertheless there are some few cases of temporary amaurosis occasioned by effusion of blood into the interior of the eye, as also between its coats, in which the prognosis is much more favorable, as it sometimes happens that vision is restored in proportion as the blood is absorbed. In general, however, the surgeon ought to be very guarded in holding out much hope to his patient in this species of amaurosis, as of all amaurotic cases, except those arising from organic changes of the eyeball, it is amongst the most unpromising.

*Treatment.* The treatment of amaurosis produced by concussion of the eyeball must be regulated by existing circumstances. In general, however, immediately after the accident, it will be found necessary to abstract blood, both locally and generally, and also to exhibit mercury. The extent and length of time that these are to be persisted in, can only be determined by the appearance of the patient. If after a fair trial of these means no benefit results, a contrary plan must be adopted, and tonics and stimulants exhibited; treating the case, in fact, as a genuine paralytic affection of the retina. The sulphate of quinia and the carbonate and tincture of the muriate of iron, may be tried in small doses. Much more advantage will, however, be derived from the use of that class of medicines denominated stimulants. These may be used either internally or externally. It will be found, however, that stimulants applied externally are much more advantageous than when used internally. Repeated blistering is perhaps the best stimulant that can be applied externally, and the nearer that they are to the affected eye, the more likely they are to be serviceable. The embrocation used in case second, I have also found of considerable use. Of electricity I cannot speak, never having seen it used. Strychnia, in consequence of its utility in other paralytic affections of the body, has been lately much tried in amaurosis, and apparently with advantage. I have given it a fair trial in several cases, and am sorry that it did not, in my hands, prove of any advantage; but perhaps I have not had a sufficient number of cases under trial to enable me to decide on its claims.

It too often, however, happens that amaurosis produced by concussion is very little, if at all, benefited by any of the above measures, the patient remaining during life perfectly amaurotic.

Species V. *Amaurosis from extravasated blood.* It may be supposed by some that this does not properly form a new species. and that it ought to have been brought in under our second. From this opinion, however, I differ, as the symptoms and changes produced in it are very different from those of the second species. I do not intend to include under this species the effusion of blood into the interior of the eyeball amongst the tunics or humours, as that has been treated of under the fourth class, but simply those cases in which blood has been effused either on the optic nerves or thalami. This extravasation may be the result of a direct injury received on the head, or of the spontaneous rupture of a blood-vessel within the brain. Violent exertion in those predisposed to apoplexy, or the sudden suppression of any accustomed discharge, frequently gives rise to the rupture of a blood-vessel within the brain, without actually occasioning that disease, but sufficient to produce amaurosis. A medical friend of mine informed me of a case in which amaurosis was instantaneously produced during the exertion of rowing a boat. The following is a well-marked case of amaurosis from rupture of a blood-vessel within the brain. John Turnbull, æt. fifty-five, coal-weigher, consulted me, in October 1829, on account of the sudden loss of the sight of his right eye. Stated, that two days previous to application, whilst attending to his business, he was suddenly seized with giddiness, and slight pain in his head, with the feeling of a snap, as if something had given way. From the immediate effects of these he recovered in a few minutes, but found that he was unable to elevate the right upper eyelid; and that when he had done so with his fingers, he saw only the half of objects, and that very indistinctly. He was assisted home, where he remained quiet for two days; but finding that he did not improve, came to consult me. On looking at him, the first thing that attracted my attention, was the upper eyelid hanging down over the eyeball, so as completely to conceal it. He had no power whatever over it. On examining the eyeball, it appeared perfectly sound. The pupil was very much contracted and immoveable, being as small as a pin-head; did not dilate with belladonna. Stated, that three years previously, he had a slight paralytic shock, of which he recovered, and that he had been subject to hæmorrhoids, and strongly suspects this attack to have been brought on by their getting better. Bowels were also confined.

Little difficulty in general is experienced in ascertaining the nature of this species of amaurosis. The sudden supervention of the disease, with the feeling of pain in the head, together with the partial or complete paralysis of some part of the body, at all times renders it exceedingly easy to be distinguished. The appearance of the eyeball is also often indicative of the nature of the disease.

Thus, paralysis of the levator palpebrarum, is nearly a constant occurrence, so that the upper lid hangs down over the eyeball, and cannot be elevated, except with the fingers, as also that of the abductor muscles. Strabismus and diplopia are also sometimes instantaneously produced in this species of amaurosis in eyes whose vision before the attack was perfect. The appearance of the pupil is almost characteristic of the disease, being contracted almost as small as a pin-head, and immoveable.

*Treatment.* The treatment of amaurosis from extravasated blood, consists in avoiding all those causes which are likely to give rise to a fresh attack of the disease, and in producing the absorption of the already effused blood. When the attack has actually come on, if the patient be strong and plethoric, and there is reason to apprehend a recurrence of the hemorrhagy, blood ought to be abstracted from the arm, as well as from the temples by cupping. The patient must be put on the antiphlogistic regimen, and confined to bed. The head ought also to be shaved, and cold evaporating lotions kept constantly applied. If, however, there is reason to suppose that the hemorrhagy is checked, we must then endeavour to promote the absorption of the effused blood. The application of blisters, and the exhibition of mercury, are the most likely means to effect this desirable end. Blisters ought to be applied on the half of the head on the affected side, and a constant discharge maintained from the surface by issue ointment. They ought also to be applied on the forehead, and when the upper lid is paralyzed, on the ramifications of the portio dura of the seventh pair of nerves: covering the blistered surface with strychnia, when the upper lid is paralyzed, has lately been strongly recommended. It is sometimes, however, impossible, by all our applications, to restore the power of the levator palpebrarum; and it then becomes a question, whether or not a small piece of the paralyzed lid may not be cut away to uncover the eyeball. Should this be determined on, it is easily accomplished with Daviel's forceps, and a pair of curved scissors, as in the common operation of entropion, from relaxation of the integuments.

Species VI. *Amaurosis from irritable conjunctiva.* I have had occasion to witness this species of amaurosis very frequently. It usually commences with more or less intolerance of light, and copious discharge of tears, the eye appearing to swim in them. At other times, however, the eyeball feels drier, with the sensation of particles of sand grating against it. On examining the eyeball, there is little or no vascularity, being only occasionally traversed with a few blood-vessels. The tarsal conjunctiva participates much more in the affection, being more vascular than natural; the meibomian secretion is also vitiated; the eyelids sometimes gluing together in the morning. Vision is not so much disturbed during day, as it is towards night, when exposed to the light of a candle, or strong fire. It then becomes much obscured; the patient either seeing objects double, and altered in position, or surrounded with a halo of different colours, which cannot be got rid of until the eye has been shut for a few minutes.

During the summer of 1829, I had an opportunity of witnessing the most marked example of this affection. The patient, a lady about 35 years of age, consulted me on account of her eyes, which she said were very weak, being unable to use them at night. She was very apprehensive of becoming blind, which was probably increased, from the circumstance of an aunt of hers being blind, and living in the house at the time. She stated that, for many months, she had been unable to use her eyes for any length of time; that they felt weak, and were constantly bathed in tears, whenever she used them. At the same time, she saw objects very indistinctly, and constantly surrounded with a halo. On examination, the eyeballs were not inflamed; there were, however, a few vessels of the conjunctiva filled with red blood, but the tarsal conjunctiva was considerably redder than natural. She complained also of an occasional pain in them, which, however, only existed when she had been using them for any length of time.

Another well-marked case presented itself to me at the time that I was drawing up this paper. February 7th, 1830, Mr. L. æt. twenty-two, consulted me on account of an affection of his eye, which came on ten days previous to application. Complained of being unable to see objects distinctly, particularly light coloured ones. Of this he was most sensible at night, when the room was lighted, at which time, if he looked at a gas-burner, he saw two, one of them being in its natural position, and the other a little to its side. That, if he persisted in using his eyes, the object became enveloped in a halo of various colours. The eyeball felt dry and uncomfortable, as if a particle of sand was grating against it. He had no pain in the eyeball. This patient was ordered to insert between his eyelids, every night at bedtime, a piece of the unguent. auratum, to avoid using his eyes as much as possible, and to take an alterative powder.

Feb. 10th. Three days after application, he reported himself to be perfectly well. The eyeball felt comfortable, and he saw objects in their natural position.

The exciting causes of this species of amaurosis are over-exertion of the eye in reading, writing, or sewing, especially by candle light, and more particularly in those of a strumous habit of body.

*Prognosis.* The prognosis of amaurosis from irritable conjunctiva, is always favorable, and the disease is rather to be regarded as a source of annoyance than as any serious loss of vision, and never, I believe, proceeds to complete amaurosis.

*Treatment.* The treatment of amaurosis from irritable conjunctiva, must consist in avoiding all over-exertion of the organ, and in the occasional use of stimulating ointments. For this purpose the eye should be protected by constantly wearing a shade of a green or black colour, and the patient, for the time, should sedulously avoid every employment which requires much use of it. The various mercurial ointments will be found of much advantage, and indeed, in many cases, after their occasional use, all the symptoms disappear, the patient recovering the perfect use of his eye. Much advantage will also be derived from the use of stimulating lotions. The muriate of mercury, or sulphate of zinc, in the proportion of a grain or two to the ounce of water, with a little rose water, is not only a grateful, but highly beneficial application. I have never found it necessary, in this species of amaurosis, to abstract blood either by cupping or leeching; and, indeed, I suspect that they would in general be injurious. Blisters are advantageous, and ought therefore never to be neglected; they should be applied behind the ears, or, what is still better, on the nape of the neck.—*Extract from the Glasgow Med. Journal.*

---

*Remarks on the Cure of Strangulated Inguinal Hernia by the Taxis; being the substance of an Essay read before the Glasgow Medical Society.* By GEORGE M'LEOD, Esq. Surgeon, &c.

In 1803, I was requested to visit a shoemaker, in Dempster street, who was labouring under strangulated inguinal hernia. The tumor occupied the scrotum to its very fundus, and had been irreducible for several hours. I determined on giving the taxis a very full trial; actuated in this, partly from having witnessed the most insignificant fingering called by that name, and partly from analogical reasoning. Cold applications were used for a short time; after which I proceeded to compress the tumor with both hands, and continued to do so, keeping up a steady pressure, with little or no remission, for nearly half an hour; at length a gurgling noise was perceived under the hands, and the protruded parts slipped into the abdomen. My patient suffered no inconvenience afterwards, and was next day at his work.

This case made on me a lasting impression. The force used was very great, as well as the length of time during which it was applied.

The sufferings of the patient while under the operation were inconsiderable, and immediately after it they were all gone. Following up this most important fact, I have ever since pursued a similar mode of treatment, and my success has been more than equal to my most sanguine expectations. During these last twenty-two years, twenty cases have fallen into my hands, and complete success has crowned my efforts in all of them, with the exception of one case, where the intestine adhered to the sac, and where the complete return of the protruded parts was impossible.

The method pursued by me is very simple. The patient is placed on his back, his knees elevated so as to relax the muscles as much as possible, the tumor is grasped at its greatest diameter by the right hand, whilst with the left the neck of the tumor is firmly supported and compressed. This last part of the operation, performed by the left hand, I deem of the utmost importance; it prevents the tumor from spreading out in a lateral direction, and consequently prevents it from doubling up over the external ring. The compression then is to be kept up by the right hand in a steady and gradually increasing manner, and not performed by jerks. If the strangulation have existed for several hours, the operation will seldom succeed in a shorter space of time than fifteen minutes, and in large herniæ a much longer space is often required; I have in such cases continued the compression from one to two hours. The difficulty of replacing the parts when the tumor is very large, appears to arise from the difficulty of grasping the tumor, and consequently the additional assistance of one or both hands of another person becomes necessary.

In detailing a few more cases, illustrative of the importance and safety of what has been nicknamed here by a learned Professor, "the two-horse-power mode of curing strangulated hernia," I shall confine myself chiefly to those cases where I was favored with the assistance or presence of my professional brethren, and thus remove, as far as is in my power, any opportunity for cavilling or doubting the facts.

A poor woman, who earned her livelihood as a washerwoman, had for many years laboured under rupture. For a great length of time she had never required assistance in replacing the protruded parts. The tumor had been gradually enlarging, and when I was called to see it, in a state of strangulation; it reached to within a hand-breadth of the knee. It had been in this irreducible state for a considerable time. After long continued attempts to return the parts by my usual mode of compression, I failed: I failed in compressing the tumor properly, on account of its enormous size; I could not compress both the body and neck at the same time, although I had recourse to the contrivance of placing the tumor on my knee, and thus supporting the back part of it. I ordered a drachm of tobacco leaf to be infused in twelve ounces of hot water, and to be administered as a clyster. Nausea and retching soon followed, and, on renewing my efforts whilst she was in this state, the tumor began to recede, and returned into the abdomen with the usual gurgling noise. The force was here continued for nearly two hours, and yet so little inconvenience did she experience afterwards, that, on my visit next day, I found her engaged at her usual occupation.

About two years after this, I was requested by my friend Mr. Harry Rainy, to visit this same person, who was again in a similar situation. The tumor was perhaps rather larger than formerly. The tobacco was administered, and the taxis persevered in again for almost a similar length of time, before we were successful. After reducing the tumor, I examined the opening through which the parts protruded, and found it so large that the points of three fingers could be pushed into it. She recovered rapidly from this attack: indeed I believe she experienced less inconvenience from that operation than did the operators; for I must confess, that my hands ached and were tremulous for two days afterwards.*

This poor woman became a third time similarly affected. The large hernial tumor again could not be returned; she had sickness, vomiting, and pain of abdomen, as well as of tumor; yet in this state she walked to the Infirmary, where, of course, the operation by the knife was performed, and when (I might almost say, of course,) she died.

A carter of the name of Howie, who resided in Mitchel street, had long laboured under reducible hernia, and from its having been almost constantly down, the herniary tumor had acquired an enormous size. From some circumstance or other, strangulation at length occurred, and I was requested to visit him. My first attempts, although very determinedly persevered in, proved unavailing, but after administering the tobacco clyster, and calling in the aid of an assistant to compress the body of the tumor, whilst the principal part of my own exertions were used in compressing the neck, I succeeded, but not until more than an hour had been exhausted in our united exertions. No bad consequences followed; he was free of pain next day, and made an excellent recovery.

Some years afterwards, I was again desired to visit this same man, but being particularly engaged at the time, it was upwards of an hour before I saw him, when I found Mr. Hood in attendance. I explained to him the plan of treatment by which I had formerly succeeded, and he at once agreed to a similar line of treatment. The tumor was now of a much larger size: it extended almost to the knee-joint, and could not by any means be grasped by two hands. After the tobacco clyster was administered, Mr. Hood, with the assistance of two carters, applied compression to the tu-

* Mr. M'Leod possesses the qualification which Celsus held to be indispensable to a surgeon, the "manus strenua," in a very uncommon degree. We deem it necessary to mention this circumstance for the sake of such of our readers as have not the pleasure of knowing Mr. M'Leod, that they may be able to appreciate the degree of force implied in the statement above.—Ed. *Glasgow Med. Journal.*

mor, whilst I applied pressure to its neck. A full hour was expended under the most unremitting exertions of the four individuals already mentioned, before the displacement was overcome, and yet our patient recovered without a bad symptom.

Sometimes cases occur where portions of omentum fall down, and, as little constitutional irritation follows, no attention is paid to the complaint. Adhesions are apt to form between the protruded omentum and sac, and the tumor consequently becomes irreducible. Intestine may afterwards follow this track and become strangulated. Such complications are very perplexing. We may reduce the protruded intestine and remove the danger, and yet a puzzling tumor may remain. - Of this variety, the following case appears to be an example.

I received a message from the late Dr. Baird, to visit, along with him, a herniary patient, in the Gallowgate; but on going there, instead of finding the Doctor alone with his patient, I found a number of medical practitioners deliberating on this case of strangulated inguinal hernia. The taxis had been tried, and although some of the gentlemen said they had felt part of tbe tumor give way, from the particular noise which it made, yet the poor man complained as much as ever. After applying my hands steadily and firmly to the tumor for some considerable time, I distinctly felt part of it slip in, when the patient declared, of his own accord, that he felt quite relieved. Some swelling could still be perceived, which had the doughy feel of omentum, and which could not be reduced. From this he felt no uneasiness however, and without further treatment he recovered rapidly.

The late Dr. George Monteath on one occasion requested me to assist him in the performance of the operation for strangulated hernia. He said the taxis and all other means had been tried in vain, and was of opinion that no time ought to be lost in relieving the stricture (as it is universally called,) by the knife. I could not resist the desire I had to compress the tumor in my usual way, and after a short perseverance, the protruded parts slipped up under my hands, to the no small mortification of at least the Doctor's pupils; for, with students and junior practitioners, the knife is every thing.

After these plain statements, I feel that it would be quite out of place to go over the different opinions and plans of treatment recommended by different men. It may be gathered from the sketch just given, that I do not believe that there exists any thing entitled to the name of stricture, however startling this declaration may appear, but that the cause alone why the protruded parts cannot be easily replaced, is the extreme distention by their contents, wind, &c. in the first instance, and enlargement in the second by fulness of vessels. I hinted, at the very commencement of these remarks, that I had been partly led by analogical reasoning to the line of treatment which I have uniformly followed for many years. The cases bearing some analogy to strangulated hernia, and which had made an impression on my mind, were those of paraphymosis. Here the stricture never becomes greater of the foreskin, but the protruded glans becomes larger, and the cure, although often attempted by dividing the stricture, is best and most easily effected by compressing the glans till it is reduced to its smallest natural size, when it very easily returns within the prepuce.

An Irish practitioner, of the name of Geohagan, in the year 1810, is the first authority I meet with who has taken any thing like a correct view of the nature of this affection. He says, "the only indication is to reduce the hernia to the size at which it had been previous to its having been strangulated, and, in furtherance of this intention, all our efforts should be directed to the removal of the air and the inflammation, and the surgeon should discharge from his mind every idea of pushing back the hernia."

Although Sir Astley Cooper is admitted to be, by the profession in general, the very highest authority on the subject of hernia, yet, in a report of one of his lectures in the Lancet, he is made to say, "that it is of no use to make pressure on the hernia with a view of emptying the intestines of their contents. This is an erroneous principle, for the contents of the intestines are very rarely the cause of stricture." It is impossible for me to explain in a clearer way my opinions on this subject, than here to declare, that they are diametrically the reverse of those of Sir Astley Cooper, given in the above quotation. The herniary tumor ought to be emptied, as one empties a gum elastic bottle.

It is but due to Mr. Lawrence to state, that although not very explicit about the mode of applying pressure in cases of strangulated hernia, he recommends a longer perseverance in the taxis than the most of authors on this subject do, and he even states, "that success will often be obtained by exerting a general pressure on the whole surface of the tumor."

---

### MISCELLANEOUS.

*Mortality of Infants.* According to recent researches made by Quetelet and Lobato, it appears that, out of 20,975 children born within the space of six years, 1044 died within the month, 390 in the course of the second month, 221 in the third, 185 in the fourth, 166 in the fifth, 156 in the sixth, 162 in the seventh, 152 in the eighth, 140 in the ninth, 153 in the tenth, 142 in the eleventh, 140 in the twelfth: total, 3051. Thus giving a considerable preponderance of mortality during the earlier months.—Dr Carus, *Compendium of Gynæcology.*

